# Finite Element Analysis of Elastoplastic Elements in the Iwan Model of Bolted Joints

**DOI:** 10.3390/ma15175817

**Published:** 2022-08-24

**Authors:** Sheng-Ao Wang, Min Zhu, Xin Xie, Biao Li, Tian-Xi Liang, Zhao-Qun Shao, Yi-Long Liu

**Affiliations:** College of Nuclear Science and Technology, Naval University of Engineering, Wuhan 430000, China

**Keywords:** elastoplastic elements, Iwan model, bolted joint, mixed-mode loading

## Abstract

The Iwan model is composed of elastoplastic elements and is widely used to represent the stiffness degradation of bolted joints under mixed-mode loading (normal and tangential loading). The latest static methods of parameter identification established the relationship between the elastoplastic elements and the contact pressure under normal loading. Under mixed-mode loading, the parameters of the Iwan model are dynamic for the evolution of contact conditions. Therefore, static parameter identification methods are not suitable for the dynamic Iwan model. A new technique was proposed to identify the parameters of the elastoplastic elements in this paper. Firstly, several different finite element models were established. The influence of the contact method and the thread structure were analyzed, and a reliable and efficient bolted-joint modeling method was proposed. Secondly, the evolution of contact conditions was studied. The dynamic elliptical contact model and the ellipticity discrete method were proposed. Finally, the residual stiffness of the Iwan model was analyzed to establish the mapping between the residual stiffness and the bending of the screw. The results can provide a technique for identifying the parameters of the dynamic Iwan model.

## 1. Introduction

Connection structures are usually applied in complicated systems. They mainly include: a movable connection structure and a fixed connection structure. The movable connection structure is generally a hinge of various forms. The fixed connection structure includes welding, riveting, bolted joints, adhesive connections, and stop ring connections, etc. [1]. Bolted joints are widely used in complex equipment for simplicity and convenience. Bolted joints mainly transfer loads via connection interfaces. Stiffness degradation caused by the interface slip seriously endangers the functionality and safety of the structure. Therefore, a theoretical model that can characterize the stiffness degradation of bolted joints is necessary.

Experiments are essential for theoretical modeling. Ungar [2] studied the sliding and damping of the bolted interface and determined the damping through the decay rate of the dynamic response. Segalman [3] designed the BMD device to reduce the interference of the unnecessary bolted surfaces and obtained the power-law relationship between energy dissipation and load amplitude. Ito et al. [4] used ultrasonic to study the pressure distribution on the bolted surface and found that the roughness affects the pressure distribution. Mantelli et al. [5] applied a pressure-sensitive film to measure the pressure distribution of bolted joints and analyzed the applicability of various pressure distribution models. However, there is no feasible apparatus to measure the evolution of contact conditions under mixed-mode loading. On the basis of experiments, many theoretical models have been proposed such as the Iwan model [6], LuGre model [7], Valanis model [8], 4-stage shear model [9], and Lu model [10], where the Iwan model is widely used for simplicity. To characterize the residual stiffness, Song et al. [11] modified the constitutive model of the Iwan model. Segalman et al. [12,13] proposed a reduced-order model based on the Jenkins element of the Iwan model. Li et al. [14] proposed a double-pulse density function (DF) of the Iwan model. The disadvantage of the modified Iwan models above is that the parameters of the DF are fitted by degradation experiments.

Li et al. [15] proposed a new method for solving the DF based on the Hertzian pressure distribution. Zhao et al. [16] proposed a technique based on the linear pressure distribution to solve the DF. The commonality of both methods is the correlation of the interfacial friction shear stress with the critical sliding forces of the Jenkins elements in the Iwan model. Both the Hertzian and the linear pressure distribution ignored the evolution of the contact evolution under mixed-mode loading.

The finite element method (FEM) is widely used in bolt analysis and has been proven to be very helpful in understanding the phenomenon observed in the experiment. Belardi et al. [17] proposed a modeling method for multi-bolt joints with a user-defined finite element and validated the model experimentally. Chen et al. [18] studied the tightening behavior of bolted joints with FEM and experiments. However, various models have been established such as the 2D model [19] and 3D model [20], etc. [21,22,23,24,25]. The analysis results are inconsistent due to different modeling methods.

The aim of this study is to develop a technique to solve the DF of the Iwan model under mixed-mode loading by FEM. We intend to provide a dynamic Iwan model and establish the relationship between the elastoplastic elements and the physical model. In Section 2, we introduce the Iwan model and the static method to solve the DF. In Section 3, we propose the method to establish a reliable bolted-joint model and the technique to associate the elastoplastic elements with the evolution of contact conditions. In Section 4, the research results are discussed, and future research directions are highlighted.

## 2. The Iwan Model and the Microslip Friction Modeling Approach

### 2.1. The Iwan Model

**Figure 1:**
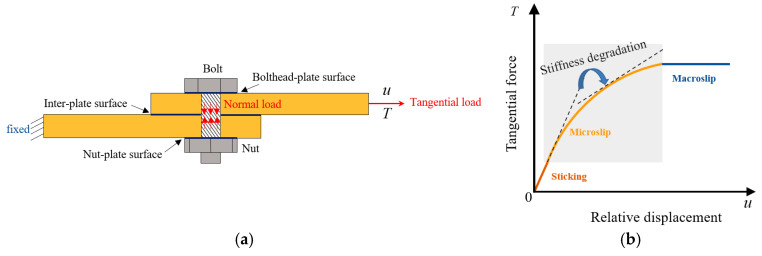
The stiffness degradation of the bolted joint under mixed-mode loading: (**a**) the mixed-mode loading, (**b**) the backbone curve.

Bolted joints are usually subjected to mixed-mode loading, as shown in Figure 1a. The lower plate is fixed, and the upper plate sustains the tangential load T. The upper plate will slide in the direction of the tangential load and slipping will occur at the inter-plate surface. Extract the tangential force T and relative displacement u to plot the backbone curve, as shown in Figure 1b. Let η represent the ratio of the sliding area to the contact area on the inter-plate surface, and the backbone curve can be divided into three parts: sticking (η=0), microslip (η∈(0,1)), and macroslip (η=1). During microslip, the stiffness undergoes a significant nonlinear degradation. The classic Iwan model can reproduce this degradation process well.

**Figure 2:**
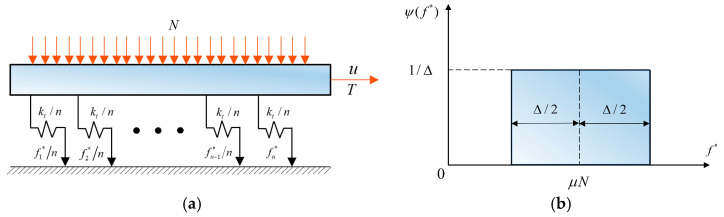
(**a**) The classic Iwan model. (**b**) Density function of the critical sliding force.

The classic Iwan model is composed of n Jenkins elements, as shown in Figure 2a. The Jenkins element is composed of a spring with stiffness $k_{t}/n$ and a friction resistor with critical sliding force $f_{i}^{*}/n$ in series. A Jenkins element is an ideal piecewise unit which can reproduce either slip or stick [26]. For simplicity, the density function $\psi (f^{*})$ in the classic model is uniform, as shown in Figure 2b, where Δ is the bandwidth.

Once the parameters of the Iwan model have been identified, the discrete form of the backbone curve can be deduced as follows

(1) $$T=\sum_{i=1}^{m}f_{i}^{*}/n+k_{t}u(n-m)/n$$

where u is the relative displacement, $k_{t}$ is the total tangential stiffness, m is the number of Jenkins elements that yield, and n is the number of Jenkins elements. The critical sliding forces of Jenkins elements are in the order of $f_{1}^{*}/n<f_{2}^{*}/n<\cdots <f_{N}^{*}/n$.

The integral form of the backbone curve of the Iwan model is

(2) $$T=\int_{0}^{k_{t}u}f^{*}\psi (f^{*})df^{*}+k_{t}u\int_{k_{t}u}^{\infty }\psi (f^{*})df^{*}$$

where $\psi (f^{*})df^{*}$ is the fraction of the total number of elements having $f^{*}\leq f_{i}^{*}\leq f^{*}+df^{*}$.

The tangential force of the unloading process under the cyclic load with displacement amplitude A is

(3) $$\overset{↼}{T}(u)=\int_{0}^{\frac{k_{t}(A-u)}{2}}-f^{*}\psi \left(f^{*}\right)df^{*}+\int_{\frac{k_{t}(A-u)}{2}}^{k_{t}A}\left[k_{t}u-(k_{t}A-f^{*})\right]\psi \left(f^{*}\right)df^{*}+k_{t}u\int_{k_{t}A}^{\infty }\psi \left(f^{*}\right)df$$

According to the Masing rule as Equation (4), the tangential force-relative displacement relation of the reloading process can be obtained.

(4) $$\vec{T}(u)=-\overset{↼}{T}(-u)$$

**Figure 3:**
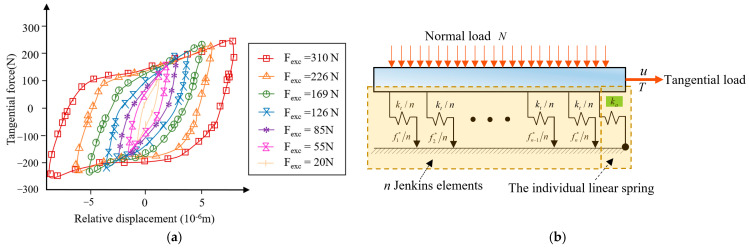
Modification of the classic Iwan model based on experiments: (**a**) experimental hysteresis loops, (**b**) the modified model.

When all Jenkins elements yield, the tangential stiffness is zero, conflicting with the residual stiffness proposed by the literature [27], as shown in Figure 3a. Song et al. [11] modified the constitutive model to represent the residual stiffness by adding a linear spring with the stiffness of $k_{a}$, as shown in Figure 3b.

### 2.2. The Microslip Friction Modeling Approach

**Figure 4:**
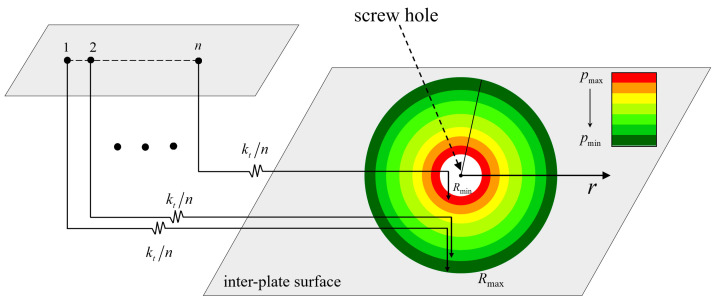
Schematic diagram of the microslip friction modeling approach.

Li et al. [15] proposed a microslip friction modeling approach by combining the classic Iwan model with a known Hertz contact pressure distribution on the contact surface. The proposed model creates a relationship between the contact pressure distribution and the DF in the Iwan model. In contrast to the classic Iwan model, the new model does not introduce additional parameters to the DF. Zhao et al. [16] applied this approach to solve the DF under the bolt preload and applied it to the Iwan model under mixed-mode loading. Both assume that the tangential load does not affect the pressure distribution on the bolted surface, as shown in Figure 4.

Assuming that the inter-plate surface pressure along the radial direction is p(r), the friction shear stress on the inter-plate surface is

(5) $$\tau_{pp}(r)=\mu p(r)$$

The area of sliding region is

(6) $$s(r)=\pi (R_{\max}^{2}-r^{2})$$

According to Equations (5) and (6), the $s(\tau_{pp})$ can be deduced. Normalize the $s(\tau_{pp})$ to $\tilde{s}(\tau_{pp})$, and take the derivative of $\tilde{s}(\tau_{pp})$ with respect to $\tau_{pp}$. Then, the DF of the friction shear force $\tau_{pp}$ is obtained, and the $\psi (f^{*})$ can be obtained based on $\tau_{pp}=\lambda f^{*}$.

## 3. Calculation and Analysis

### 3.1. Finite Element Modeling

Due to the insufficiency of the experimental method, we applied the FEM to analyze the evolution of the contact condition of the bolted joint under mixed-mode loading. Most researchers have replaced the threaded structure with bonding contact for simplicity and convergency. The feasibility of the thread simplification still needs to be verified. Therefore, the thread model and the simplified model were established and compared. The finite element model was constructed by Abaqus and Hypermesh software, and the simulations were conducted on the server (computer computing environment: Windows 10 operating system, 2.30 GHz Intel Xeon Gold 6140 CPU, and 192 GB RAM). We applied the Abaqus/Standard main solver module to solve the quasi-static problem. Abaqus/Standard is a general analysis module that uses implicit analysis methods to solve linear and nonlinear problems, such as static, dynamic, and complex multi-field-coupled analyses.

**Figure 5:**
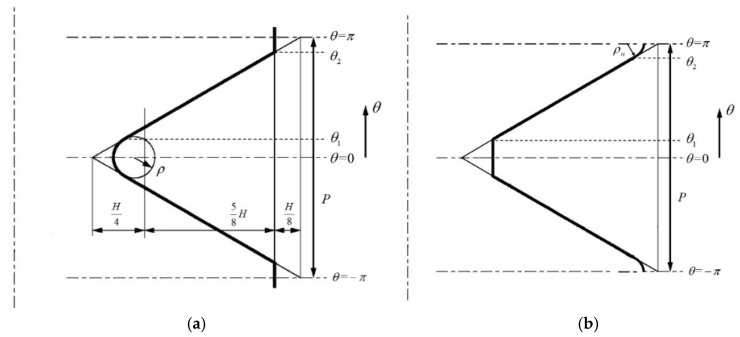
(**a**) The external thread profile. (**b**) The internal thread profile (The thread pitch P is 1.25 mm, the radius $\rho_{n}$ is 0.07 mm, the thread height H is 1.08 mm, and the radius ρ is 0.14 mm).

The bolt head is a cylinder with a radius of 6.5 mm and a thickness of 5 mm, and the total length of the screw is 35 mm. The thread is constructed according to Figure 5.

**Figure 6:**
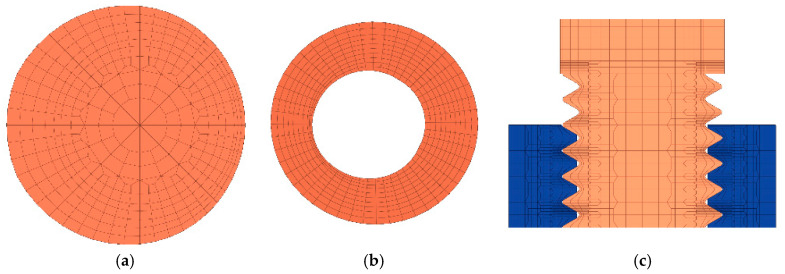
Finite element models of the bolt and nut: (**a**) cross section of bolt, (**b**) cross section of nut, (**c**) cross section of bolt and nut assembly.

The thread is applied at the nut, and the diameter of holes on the jointed plates is larger than that of the screw [16,28]. The established high-quality models of the bolt and nut are shown in Figure 6. The mesh of the bolt cross section and the hole of the nut are not symmetric with respect to the axis due to the thread lead angle of 2.8473°.

**Figure 7:**
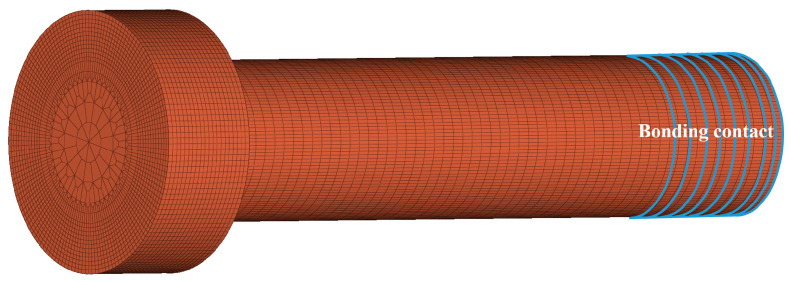
Simplified bolt model.

In the simplified model, the screw is a cylinder with a diameter of 8 mm, and the nut is a hollow cylinder. The simplified model is as shown in Figure 7. The difference between the simplified model and the thread model is that the simplified model simulates a threaded connection through bonding contact.

**Figure 8:**
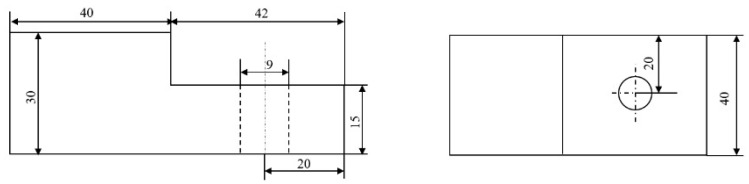
Dimensions of plates (mm).

The specific geometrical dimensions of the plate are shown in Figure 8. A gap was left to avoid interference between the bolt and the hole. According to the ISO Mechanical Design Manual, the diameter of the hole was 9 mm. There was a 0.5 mm gap between the screw and hole. Generally, the magnitude of microslip was on the order of microns, and a space of 0.5 mm was reserved enough for the microslip and macroslip of the inter-plate surface.

**Figure 9:**
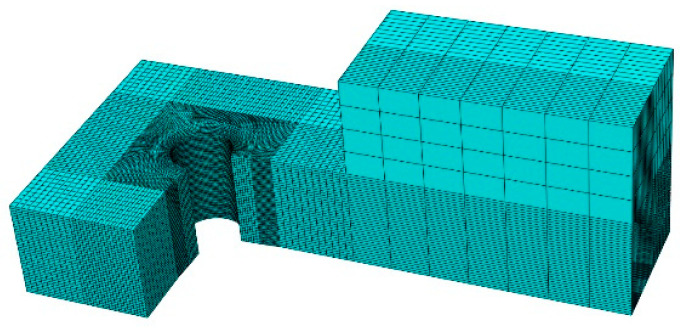
Cross section of the finite element model of the plate.

To improve the calculation efficiency and accuracy, the mesh near the bolt hole was locally refined, as shown in Figure 9. There are various element types in Abaqus, among which, linear-reduced integration elements have the characteristics of accurate displacement results and fast calculation speed and are suitable for contact analysis. Therefore, the linear-reduced integration element C3D8RH was adopted. The material of the model was alloy AISI4340, based on the literature [29], whose elastic modulus was 205.9 GPa and Poisson’s ratio was 0.3. The plastic properties were not considered because no plastic deformation of the material was found in past experiments.

**Figure 10:**
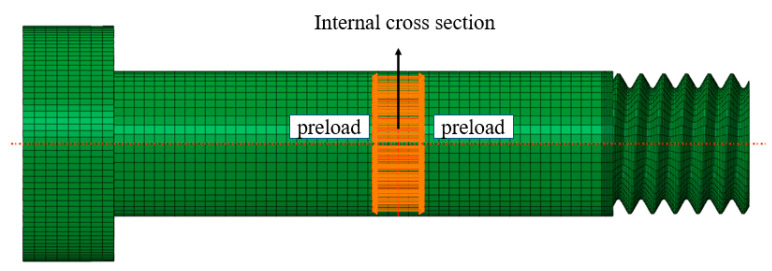
Schematic diagram of bolt preload.

The bolt preload was simulated by defining an internal section on the screw and the direction and amplitude of the preload, as shown in Figure 10.

**Figure 11:**
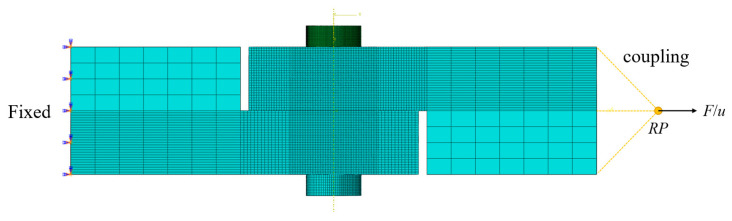
Initial and boundary conditions.

The tangential load was applied on the reference point (RP) coupled with the right surface of the upper plate, and the left surface of the lower plate was fixed, as shown in Figure 11. The friction coefficient of steel is usually between 0.42 and 0.78, and the friction coefficient of all contact surfaces in this paper was 0.6 [30].

Different bolt preload leads to different macroslip displacements. It was necessary to determine the amplitude of the preload so that the macroslip displacement was less than the gap of 0.5 mm. Through pre-calculations for both the thread model and simplified model, the macroslip displacement of the 1000 N preload was 0.0036 mm. The tangential displacement load was 0.015 mm, which could reproduce the whole microslip. The analysis steps are as follows.

Step 1: Apply a preload of 1000 N to the bolt’s internal cross section;

Step 2: Apply a tangential displacement of 0.015 mm at RP in the tangential direction.

The tangential behavior can be represented by the Coulomb law with the Lagrange multiplier or penalty method. The Lagrange multiplier method reproduces the Coulomb law but does not converge in pre-calculations for the large node displacement. Instead, the calculation with the penalty method can converge. It is necessary to analyze the feasibility of the penalty method to replace the Lagrange method.

### 3.2. Analysis of the Lagrange Multiplier and Penalty Method

**Figure 12:**
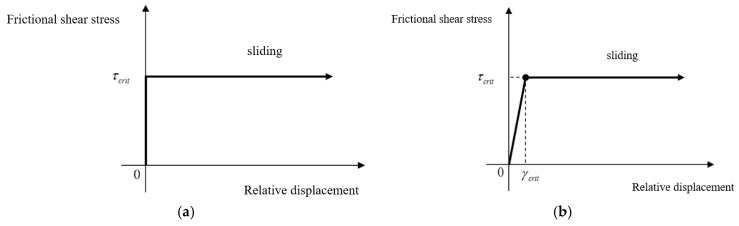
Relationship between relative displacement and friction shear stress of different contact methods: (**a**) Lagrange multiplier method, (**b**) penalty method ($\tau_{crit}$ is the critical friction shear stress, $\gamma_{crit}$ is the relative displacement of critical sliding.).

The Lagrange multiplier method enforces the relative displacement of the adhesive contact to be zero, as shown in Figure 12a, which strictly matches the Coulomb law. Due to the introduction of multipliers by the Lagrange multiplier method, the order of the equation increases, and the stiffness matrix is no longer a symmetric positive definite matrix. Solving the corresponding multipliers can accurately satisfy the constraint equations, but it will lead to great convergence difficulties because of the loss of positive definiteness.

**Figure 13:**
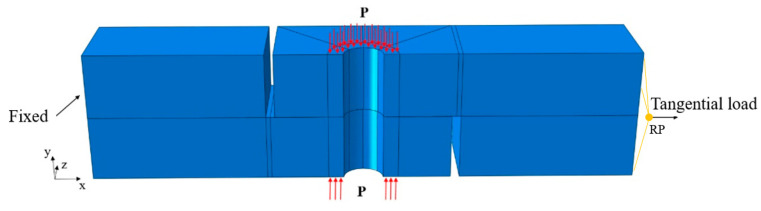
The lapped plate under mixed-mode loading.

The penalty method is an approximate method where the relative displacement of adhesive contact is not zero but follows hard-elastic behaviour, as shown in Figure 12b. It does not change the order and positive definiteness of the system matrix, and the system matrix is theoretically easier to be solved. Since both models cannot converge with the Lagrange multiplier method, an equivalent model was established to analyze the difference between the two methods, as shown in Figure 13. A uniform pressure of 200 MPa was applied to the bolt-plate surfaces to simulate the normal load (bolt preload). The tangential displacement of 0.2 mm was applied to simulate the tangential load.

**Figure 14:**
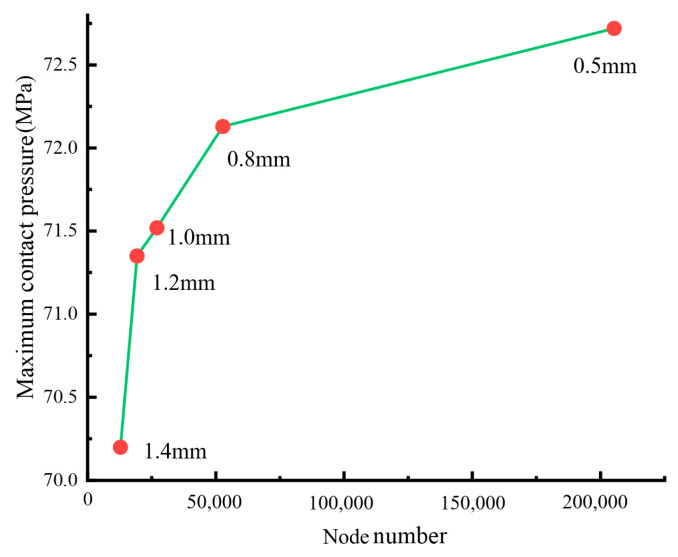
The mesh convergence curve.

To improve the confidence of the calculation results, the mesh convergence analysis was performed. The relationship between the nodes number and the maximum contact pressure on the inter-plate surface was as shown in Figure 14. When the mesh was between 0.5 mm and 0.8 mm, the maximum contact pressure was stable at 72~73 MPa. The node number of the 0.5 mm model was four times that of 0.8 mm, requiring higher computing resources.

**Figure 15:**
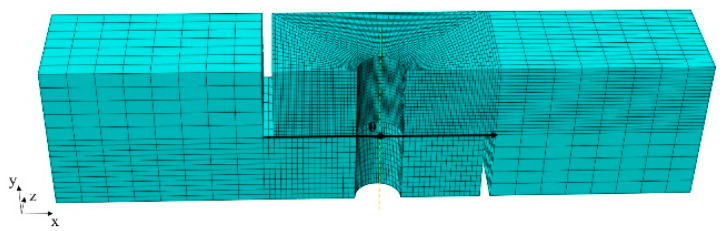
The finite element model of lapped plates.

The mesh of 0.8 mm was applied in the equivalent model. The coarser mesh may lead to node penetration from the slave surface to the master surface, resulting in poor calculation reliability. In this paper, the mesh of the master surface was refined to suppress the penetration. The grid model is shown in Figure 15, where the node number of the upper and lower plate was 44,145 and 8160, respectively.

**Figure 16:**
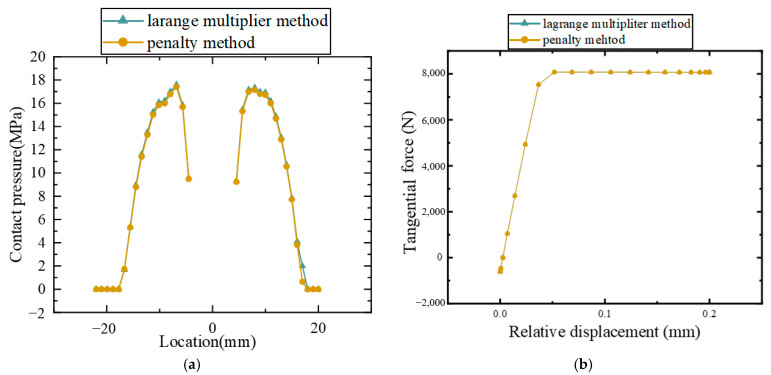
Calculation results of the Lagrange multiplier method and the penalty method: (**a**) contact pressure distribution curves, (**b**) backbone curves.

The pressure distribution along the *x*-direction of the inter-plate surface under preload and the backbone curves under mixed-mode loading are shown in Figure 16. The Pearson correlation coefficient of the pressure distribution curves was 0.99956, and that of the force-displacement curves was 0.99999. The results show that both contact methods are suitable from the point of view of providing similar results, and the Lagrange multiplier method could be replaced by the penalty method in the bolted plate model under mixed-mode loading.

### 3.3. Analysis of the Thread Model and the Simplified Model

**Figure 17:**
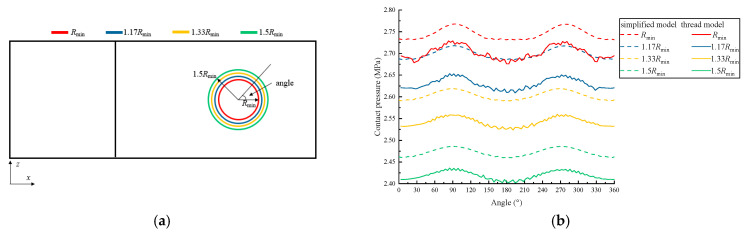
Pressure distribution on the inter-plate surface: (**a**) scanning paths on the inter-plate surface, (**b**) the pressure distribution of the two models.

We set four pressure scanning paths on the inter-plate surface, as shown in Figure 17a. The radius of the hole is represented by $R_{\min}$, and the radii of the four circles are *a*, 1.17 $R_{\min}$, 1.33 $R_{\min}$, and 1.5 $R_{\min}$, respectively.

According to the pressure distribution curves in Figure 17b, the pressure distributions of the two models show harmonic law, which is not caused by the thread structure but rather by the asymmetry of the plate. The thread structure makes pressure distribution curves of the thread model fluctuate within a small amplitude. Since the thread structure is replaced by the bonding contact in the simplified model, the normal stiffness increases, causing the pressure amplitude to be slightly larger than that of the thread model.

**Figure 18:**
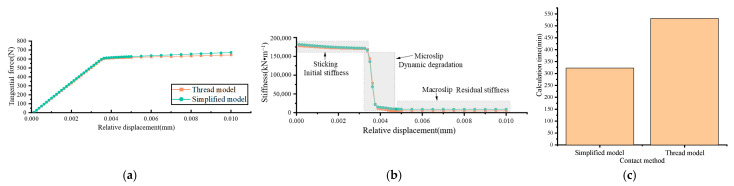
Dynamic degradation of the thread model and simplified model under mixed-mode loading: (**a**) backbone curves, (**b**) stiffness degradation curves, (**c**) calculation time.

The calculation results of the two models under mixed-mode loading are as shown Figure 18. The backbone curves of the two models coincided, and there were only slight differences in the macroslip stage. The microslip could not be clearly distinguished by the backbone curves, as shown in Figure 18a. According to the stiffness degradation curves, as shown in Figure 18b, in the simplified model, the initial stiffness and residual stiffness were higher than those in the thread model.

The degradation of bolted joints presented by the simplified model and the thread model were consistent, and the friction on the threaded surfaces was negligible. The simplified model was available to analyze the dynamic degradation of bolt joints with a balance of efficiency and accuracy.

### 3.4. Analysis of Elastoplastic Elements under Mixed-Mode Loading

The above analysis indicates that the calculation results of the simplified model were reliable. Therefore, based on the calculation results of the simplified model under mixed-mode loading, we analyzed the relation between elastoplastic elements of the Iwan model and the evolution of the contact conditions.

Under bolt preload loading, the mapping between the elastoplastic Jenkins elements in the Iwan model and the friction shear stress was established in Section 2. Such mapping is not suitable for the mixed-mode loading condition.

**Figure 19:**
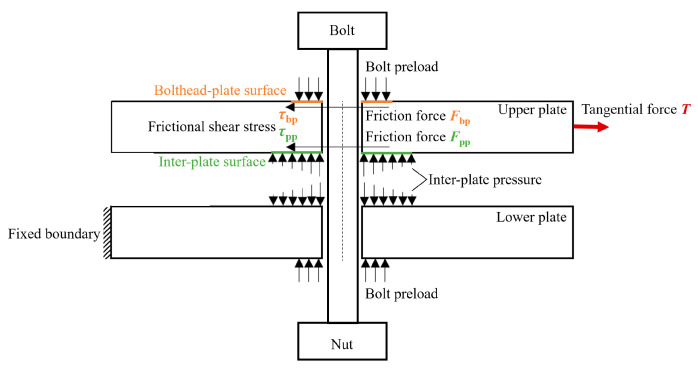
Force decomposition.

Under mixed-mode loading, the tangential force is composed of the friction force on the bolthead-plate surface $F_{bp}$ and that on the inter-plate surface $F_{pp}$, as shown in Figure 19. The tangential force T satisfies $T=F_{pp}+F_{bp}$, while the tangential force T in the Song modified model is

(7) $$T=[\int_{0}^{k_{t}u}f^{*}\psi (f^{*})df^{*}+k_{t}u\int_{k_{t}u}^{\infty }\psi (f^{*})df^{*}]+k_{a}u$$

**Figure 20:**
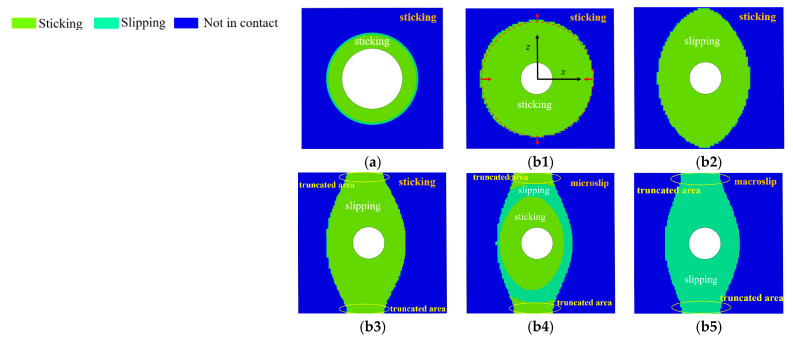
The evolution of contact conditions under mixed-mode loading: (**a**) Contact condition on the bolthead-plate surface, (**b**) contact condition on the inter-plate surface: (**b1**) *T* = 0 N, (**b2**) *T* = 228.555 N, (**b3**) *T* = 489.332 N, (**b4**) *T* = 575.232 N, (**b5**) *T* = 632.458 N.

The bolthead-plate surfaces were always in partial contact and sticking under mixed-mode loading, as shown in Figure 20a. The result indicates that $F_{bp}$ is a static friction force that cannot be solved through pressure and friction coefficient. According to Figure 20(b1), the contact area was circular under the initial bolt preload. The contact boundary shrank in the *x*-direction and stretched in the *z*-direction, as shown in Figure 20(b1–b3). When T=228.55 N, the contact boundary reached the plate boundary in the *z*-direction. When T=489.332 N, the contact boundary was truncated by the plate boundary in the *z*-direction. When 228.55 N≤T≤489.332 N, the contact condition was sticking, and the mapping was that the force sustained by the Jenkins element was smaller than the minimum critical slip force ($k_{t}u<f_{\min}^{*}$).

When 575.232 N≤T≤632.458 N, microslip occurred. The central and the truncated area remained sticking, as shown in Figure 20(b4). When the tangential force was larger than 632.458 N, the macroslip occurred, and $F_{pp}$ was equal to the product of the preload and the friction coefficient μ($F_{pp}=\mu N$).

With the increase in the tangential load T, the contact boundary of the inter-plate surface varied, which means that the DF was dynamic. The contact boundary resembled an ellipse, except for the truncated area.

**Figure 21:**
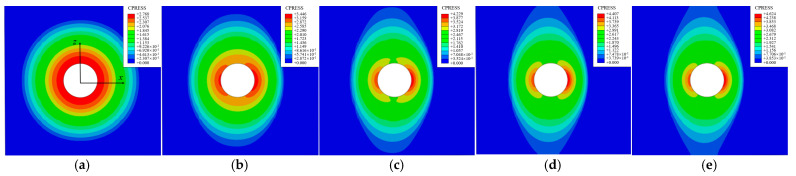
Pressure distribution on the inter-plate surface under mixed-mode loading: (**a**) *T* = 0 N, (**b**) *T* = 228.555 N, (**c**) *T* = 489.332 N, (**d**) *T* = 575.232 N, (**e**) *T* = 632.458 N.

As shown in Figure 21a, the pressure was centrosymmetric, which was suitable for the microslip friction modeling in Section 2.2. As T increased, the peak pressure increased from 2.768 MPa to 4.624 MPa. The pressure of the central area accumulated in the *x*-direction. The pressure distribution decreased from the center to the surrounding, and the pressure in the truncated area was relatively low.

**Figure 22:**
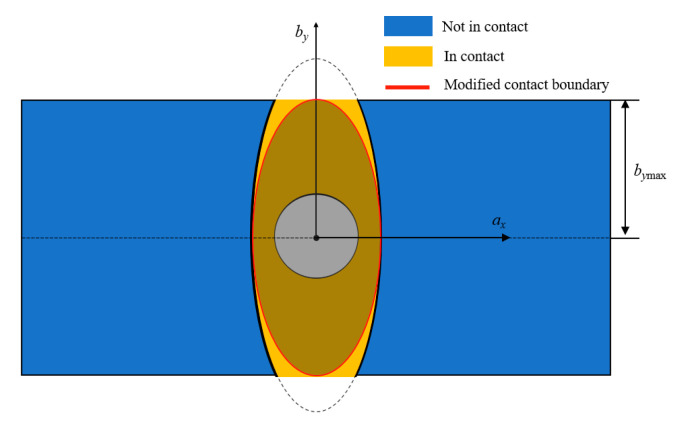
Ellipse modification in microslip.

Based on the analysis above, we simplified the contact area to an ellipse at the expense of a small amount of preload, as shown in Figure 22. The two axes of the ellipse were $a_{x}$ and $b_{y}$, and the modified area was an ellipse with the semi-major axis of $b_{y\max}$ for narrow plates. In microslip, the semi-minor axis was a dynamic value of $a_{x}^{T}$. The dynamic ellipse can be described by function as

(8) $$\frac{a_{x}^{2}}{{\left(a_{x}^{T}\right)}^{2}}+\frac{b_{y}^{2}}{{\left(b_{y\max}\right)}^{2}}=1$$

**Figure 23:**
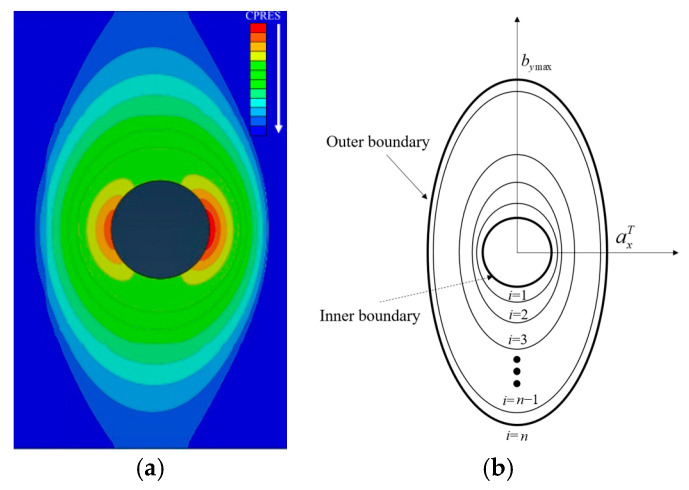
Ellipticity discrete method: (**a**) isolines of pressure on the inter-plate surface, (**b**) ellipticity discrete method.

The microslip friction modeling approach in Section 2.2 does not work on the dynamic ellipse. According to Figure 23a, the isolines have different ellipticity. The ellipticity discrete method was proposed, as shown in Figure 23b. The *i*th and the (*i* − 1)th ellipse form a sliding area of $s_{i}$.

The ellipticity of the *i*th ellipse is $\rho_{i}$, linearly increasing from 1 to $b_{y\max}/a_{x}^{T}$ with the increase of *i* from 0 to n. The ellipticity of the *i*th ellipse is

(9) $$\rho_{i}=1+\frac{b_{y\max}-a_{x}^{T}}{a_{x}^{T}n}i$$

The semi-minor axis and semi-major axis of the *i*th ellipse are

(10) $$\begin{array}{c} a_{x}^{i}=R_{\min}+\frac{a_{x}^{T}-R_{\min}}{n}i\\ b_{y}^{i}=\rho_{i}a_{x}^{i} \end{array}$$

Then the area of the *i*th sliding area is

(11) $$s_{i}=\pi (a_{x}^{i}b_{y}^{i}-a_{x}^{i-1}b_{y}^{i-1})$$

The variation law of pressure distribution in the *x*-direction is not the research content of this paper, and it is assumed to be $p_{T}(r)$. Thus, the pressure on the $s_{i}$ is $p_{T}(a_{x}^{i})$. Since the elliptical contact area assumption and the ellipticity discrete method sacrifice part of the preload, the corrected pressure distribution $p_{Tc}(r)$ is

(12) $$p_{Tc}(r)=\frac{N}{\sum_{i=1}^{n}s_{i}p_{T}(a_{x}^{i})}p_{T}(r)$$

The normal load $N_{i}$ is

(13) $$N_{i}=s_{i}p_{Tc}(a_{x}^{i})$$

The friction shear stress $\tau_{i}$ of the *i*th sliding area is

(14) $$\tau_{i}=\mu N_{i}$$

When n tends to infinite, the DF of friction shear stress Ψ(τ) is deduced by normalization and derivation. Thus, the DF of the Iwan model is

(15) $$\psi (f^{*})=\lambda \Psi (\lambda f^{*})$$

**Figure 24:**
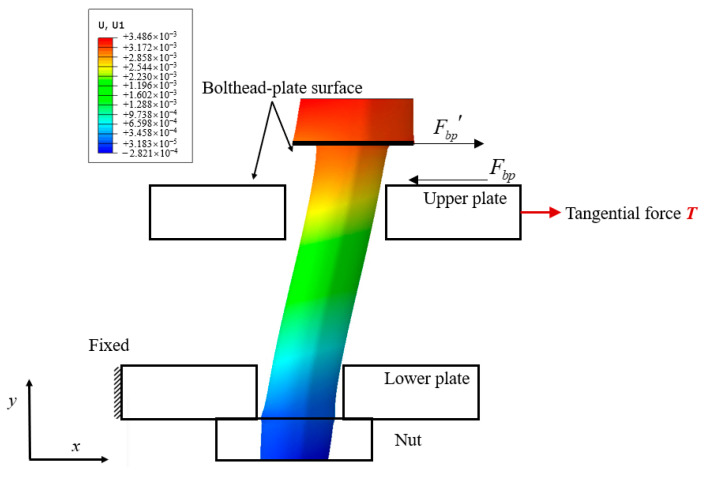
The displacement of the bolt in the *x*-direction.

Then the mapping between the friction shear stress and critical sliding force can be established. The $F_{pp}$ is the source of the $\left[\int_{0}^{k_{t}u}f^{*}\psi (f^{*})df^{*}+k_{t}u\int_{k_{t}u}^{\infty }\psi (f^{*})df^{*}\right]$ in the Iwan model. Therefore, it can be inferred that the $F_{bp}$ is the source of $k_{a}u$, and the residual stiffness $k_{a}$ is equal to the tangential stiffness of the bolthead-plate surface $dF_{bp}/du$. It is difficult to measure the variation law of static friction $F_{bp}$ under mixed-mode loading. The reaction force $F_{bp}{}^{\prime }$ acting on the bolt head is shown in Figure 24. The bolt head-plate surface and the nut-plate surface always remain sticking under mixed-mode loading, and the lower plate is fixed while the upper plate moves with the tangential load. The bolt head moves in the *x*-direction with the upper plate, and the other end is fixed on the stationary lower plate, as shown in Figure 24. Therefore, the bending stiffness of the bolt can be measured to predict the residual stiffness $k_{a}$ in the Iwan model.

## 4. Discussion

In this paper, we established a reliable finite element model and analyzed the contact and dynamic degradation of the bolted joint under mixed-mode loading. A new technique to identify parameters of $f^{*}$ and $k_{a}$ in the dynamic Iwan model was proposed. The specific conclusions are as follows.

(1) The penalty method sacrifices accuracy for convergence, but it is closer to the real situation than the Lagrange multiplier method. Through analysis, the calculation results of both methods are the same for lapped plates, and the penalty method is more suitable for the contact analysis of bolted plates.
(2) A threaded connection reduces the normal and tangential stiffness of the joints compared to bonding contact, but the reduction is negligible. Therefore, in finite element analysis, it is feasible to use bonding contact to replace the threaded connection.
(3) The contact area of the inter-plate surface changes with the tangential load under mixed-mode loading, and the contact boundary can be represented by an ellipse function. In microslip, the semi-major axis remains unchanged and the semi-minor axis is a function $a_{x}^{T}(T)$ of the tangential force *T*.
(4) On the premise of the known dynamic pressure distribution, the contact area can be discretized by the different ellipticity. The discrete method can be applied to dynamic elliptical boundaries, and the DFs of friction shear stress and critical sliding force can be solved.
(5) The residual stiffness of the Iwan model is derivative of static friction to relative displacement, and the static friction force causes the bending of the screw.

The analysis of elastoplastic elements can further improve the application scenarios of the Iwan model and develop the Iwan model from a static model to a dynamic model. These results may be useful in appropriate designs of the bolted joint under stiffness and damping criteria.

Abad et al. [21] also used the FEM to analyze the degradation of bolted joints under mixed-mode loading and identified parameters of the Valanis model. However, the relation between the similar evolution of contact conditions and the theoretical model was not established. It indicated that the Iwan constitutive model has great potential for development, but there is still much work to be done as follows:

(1) The pressure distribution law under mixed-mode loading needs to be studied, and a function needs to be constructed to characterize the pressure distribution in the non-circular area.
(2) Experiments are indispensable to verify the theory. Designing equivalent bolted plates to overcome the shortcomings of ultrasonic methods to measure pressure distribution across multiple interfaces may be a feasible technique.
(3) The mapping of the parameter $k_{t}$ in the constitutive model is still not clear, and the mapping parameter λ also needs to be studied.

## Data Availability

Not applicable.

## Nomenclature

T	tangential force	u	Relative displacement
N	normal load	μ	friction coefficient
$F_{exc}$	amplitude of excitation force	$k_{a}$	Residual stiffness
n	number of Jenkins elements	$R_{\max}$	maximum radius of contact area
$R_{\min}$	radius of the hole	$\tau_{bp}$	friction shear stress on the bolthead-plate surface
p	Contact pressure	$\tau_{pp}$	friction shear stress on the inter-plate surface
$k_{t}$	tangential contact stiffness	$F_{bp}$	friction force on the bolthead-plate surface
$p_{c}$	corrected pressure	$F_{pp}$	friction force on the inter-plate surface
s	sliding area	A	amplitude of the cyclic load
$f^{*}$	critical sliding force	$a_{x}^{T}$	semi-minor axis of the contact boundary
Δ	range of critical sliding force	$a_{x}^{i}$	semi-minor axis of the *i*th ellipse
$b_{y\max}$	semi-major axis of the contact boundary	$b_{y}^{i}$	semi-major of the *i*th ellipse
H	thread height	$\rho_{i}$	ellipticity of the *i*th ellipse
DF	density function	η	ratio of the sliding area to the contact area
$p_{T}$	contact pressure under mixed-mode loading	$p_{Tc}$	corrected contact pressure under mixed-mode loading
